# The Role of MicroRNAs and Other Molecular Biomarkers in Uveitis: Protocol for a Scoping Review

**DOI:** 10.2196/89265

**Published:** 2026-09-25

**Authors:** Raza Ray Abbas Syed, Andrea Doyle, Valeria Lima Passos, Kumail Matthew Abbas Syed, Killian Ross Walsh, Joan Ní Gabhann-Dromgoole, Conor Murphy

**Affiliations:** 1 Department of Ophthalmology Royal Victoria Eye and Ear Hospital Dublin, Leinster Ireland; 2 School of Pharmacy and Biomolecular Sciences (PBS) Royal College of Surgeons in Ireland Dublin 2, Leinster Ireland; 3 SIM Centre for Simulation Education and Research Royal College of Surgeons in Ireland Dublin, Leinster Ireland; 4 School of Medicine University of Limerick Limerick, Munster Ireland; 5 Library Services Royal College of Surgeons in Ireland Dublin, Leinster Ireland

**Keywords:** uveitis, microRNAs, cytokines, chemokines, genomics, proteomics, transcriptome, metabolomics, multiomics, biomarkers, review

## Abstract

**Background:**

Uveitis is a complex disease involving inflammation of the iris, ciliary body, and choroid. The Standardization of Uveitis Nomenclature working group has developed classification criteria for 25 of the most common uveitis entities based on a number of descriptor terms. Due to the broad range of causes of uveitis and the different treatment modalities depending on etiology, investigative aids are vital in expediting accurate diagnosis and management. MicroRNAs, small noncoding RNAs, have exciting potential as biomarkers of disease. They function to alter gene expression at the posttranscriptional level. Other biomarkers of inflammatory disease include cytokines and chemokines. With a substantial proportion of uveitis being classified as “idiopathic,” biomarker research may provide important diagnostic clues and subsequently improve patient outcomes globally. Scoping reviews represent a format for systematically searching the literature. We will use a formal framework for conducting scoping reviews and the Joanna Briggs Institute guidelines.

**Objective:**

The aim of this review protocol is to lay the foundation for identifying what biomarkers have been studied in various uveitis subtypes, including cytokines, chemokines, and multiomic data, with particular focus on microRNAs, thus guiding the selection of biomarkers that may be of interest in future research.

**Methods:**

Studies will be included if they have human participants with a defined diagnosis of a uveitis subtype who are of any age, gender, or ethnicity and from any country or health care setting; assess molecular biomarkers (including but not limited to microRNAs, cytokines, chemokines, human leucocyte antigen types, gene variants, and multiomics) using any sample type (including but not limited to blood, peripheral blood mononuclear cells, aqueous humor, vitreous humor, cerebrospinal fluid, and urine), are primary research studies, are written in English or with an English translation, and were published in any year. Studies will be excluded if they have animal participants, investigate therapeutics in uveitis only, are review articles or letters to the editor, are research in progress, or are not in English. The MEDLINE, Embase, and Scopus databases will be searched from inception to June 8, 2026. Results will be descriptively analyzed and presented in the form of a data extraction chart.

**Results:**

The proposed scoping review is currently in the screening stage. The start date for this project was January 6, 2026. The projected completion date is in October 2026. A total of 946 articles were subject to title and abstract screening using Covidence software. One reviewer screened all papers, while a second reviewer screened 20% of the papers.

**Conclusions:**

The findings from this scoping review will highlight the broad range of molecular biomarkers currently studied in uveitis and identify gaps in the field, guiding the selection of biomarkers, with a particular focus on microRNAs, for future laboratory analysis in our research group.

**Trial Registration:**

OSF Registries N5HJR; https://osf.io/n5hjr/overview

**International Registered Report Identifier (IRRID):**

PRR1-10.2196/89265

## Introduction

### Background

Uveitis is a complex disease, involving inflammation of the iris, ciliary body, and choroid [1]. The Standardization of Uveitis Nomenclature (SUN) working group has developed classification criteria for 25 of the most common uveitis entities based on a number of descriptor terms [2]. These include anatomical location, infectious etiology, systemic disease association, and whether the uveitis is eye-limited. Management of uveitis is determined by its etiology, with infectious and autoimmune mechanisms predominating [3,4]. This has important implications when determining treatment choice. Due to the broad range of causes of uveitis and the different treatment modalities depending on etiology, investigative aids are vital in expediting accurate diagnosis and management.

One systematic review and meta-analysis examining the epidemiology of uveitis found a pooled annual incidence estimate of 50.45 per 100,000 population [5]. The prevalence estimates in the same review ranged from 2 to 730 per 100,000 population. A claims-based analysis of the prevalence of noninfectious uveitis in the United States estimated prevalence of 121 cases per 100,000 adults [6]. Adults aged 20 to 50 years are most commonly affected, therefore implicating people of working age [1]. In the United States and Europe, uveitis is responsible for between 3% and 10% of visual impairment [7]. The prevalence of this disease and its association with younger patient cohorts makes rapid diagnosis and subsequent early treatment vital to preserve vision, maintain individual quality of life, and reduce economic burden. Despite this, a significant amount of uveitis remains unclassified. One study conducted in Japan of 1174 patients found that 44.9% of uveitis cases were unclassified [8]. Similarly, a study of 5791 patients conducted in Pakistan found that 33.2% of uveitis cases were idiopathic [9]. In Finland, 413 cases of anterior uveitis were analyzed, with 47% considered idiopathic [10]. The recommendations of the SUN working group split uveitis into categories based on the following: anatomical location, infectious, systemic disease associated, and eye-limited [2]. This highlights the heterogeneous nature of the disease. The pathogenesis of uveitis has been investigated through the use of rat models. These include endotoxin-induced uveitis (EIU) and experimental autoimmune uveoretinitis (EAU) [11-14]. EIU is created via systemic injection of lipopolysaccharide, and EAU is produced by systemic injection of retinal S antigen.

Uveitis presents differently depending on the predominant location of inflammation and etiology. Anterior uveitis causes pain, red eye, photophobia, and blurred vision. Intermediate uveitis presents with painless floaters and blurred vision. Posterior uveitis may present similarly to this, as well as with nyctalopia and photopsia in some cases [1]. Diagnosis is made largely via clinical examination, which may reveal the anatomical location of inflammation and identify clinical signs that provide diagnostic clues. Investigations include optical coherence tomography to detect posterior segment involvement with or without cystoid macular edema [15]. Fundus fluorescein angiography, indocyanine green angiography, and fundus autofluorescence may be useful in detecting active posterior segment lesions [15]. Serological tests are often used in severe, bilateral, or recurrent cases [16]. Treatment is directed at the cause and often includes topical, periocular, intraocular, or systemic corticosteroids, or immunosuppressive therapy [17].

The Food and Drug Administration and National Institutes of Health published the BEST (Biomarkers, Endpoints, and Other Tools) resource in 2016, defining various biomarker types. Diagnostic biomarkers are used to detect the presence or absence of disease [18]. MicroRNAs were first isolated from the *Caenorhabditis elegans* nematode in 1993 by Lee et al [19]. They can be defined as small, noncoding, endogenous, conserved RNA molecules ranging from 17 to 25 nucleotides in length. They regulate gene expression at the posttranscriptional level [20,21]. They possess roles in various biomolecular functions, from apoptosis to cell proliferation, metabolism, and tissue differentiation [22-24]. Other biomarkers of inflammatory disease include cytokines and chemokines. Optimal traits of an ideal biomarker include high sensitivity, early detection, high stability, accessibility, and translatability [25].

The role of microRNAs as biomarkers of various pathologies is well documented. Long et al [26] investigated the expression levels of miR-30a, miR-126, and let-7b in patients with ischemic stroke compared to controls. miR-30a and miR-126 were found to be downregulated in all the patients with ischemic stroke until 24 weeks after the event, and let-7b was differentially expressed depending on ischemic stroke subtype compared to controls [26]. MicroRNAs have been well characterized in the study of breast cancer. Khalighfard et al [27] demonstrated upregulation of miR-21, miR-10b, and miR-155 in patients with breast cancer compared with healthy controls. With regard to inflammatory conditions, specifically uveitis, microRNAs have been postulated as biomarkers of disease. Asakage et al [28] conducted a comprehensive serum microRNA analysis using untargeted techniques to identify dysregulated microRNA expression in patients with uveitis with Vogt-Koyanagi-Harada (VKH) syndrome, Behçet disease, and ocular sarcoidosis, compared to control. miR-4708-3p, miR-4323, and let-7g-3p were identified as the best predictors for Behçet ocular sarcoidosis, and VKH syndrome, respectively [28]. Pilson et al [29] investigated the role of microRNAs in human conjunctival epithelial cells in patients diagnosed with primary Sjögren disease. miR-744-5p expression was found to be elevated compared to controls. Pellino3, a negative regulator of inflammation, was validated as being a target of miR-744-5p. This was significantly reduced in human conjunctival epithelial cells of patients compared to the controls [29].

Cytokines and chemokines are biomarkers of interest when studying inflammatory diseases. Systemic lupus erythematosus (SLE) is characterized by widespread inflammation and tissue damage [30]. One study found that patients with SLE had higher plasma concentrations of the chemokines IP10, RANTES (regulated upon activation, normal T-cell expressed and secreted), MIG (monokine induced by gamma interferon), MCP-1 (monocyte chemoattractant protein 1), and GRO-alpha (growth-regulated oncogene-alpha protein), as well as the cytokine interleukin-18, compared to controls [31]. With regard to uveitis, Lacomba et al [32] showed elevated aqueous and serum levels of interferon-γ and interleukin-2 levels compared to controls. This highlights their potential as diagnostic biomarkers of uveitis [32].

Genomics pertains to the study of the entire genome and the data generated from it [33]. Transcriptomics refers to the study of transcripts or messenger RNAs and noncoding RNAs [34]. Proteomics involves the analysis of proteins produced or modified by an organism. Metabolomics examines molecules involved in metabolic processes [35]. These form the strata of the overarching term, multiomics. Combination analysis of multiple types of omics data allows for the generation of inferences that are unavailable when studied alone [36]. Multiomics has been applied to the study of ocular diseases. Arakawa et al [37] demonstrated 2 susceptibility loci for age-related macular degeneration: TNFRSF10A-LOC389641 on chromosome 8p21 and REST-C4orf14-POLR2B-IGFBP7 on chromosome 4q12. Senabouth et al [38] used induced pluripotent stem cells from patients with geographic atrophy secondary to age-related macular degeneration to carry out multiomics analysis, revealing 3911 differentially expressed genes compared with controls. Proteomic analysis identified 234 proteins with differential expression in the geographic atrophy cohort compared to controls [38]. Mitochondrial transcripts were upregulated compared to controls, as well as various metabolic pathways, such as the ATP (adenosine triphosphate) and NAD/NADH (nicotinamide adenine dinucleotide/nicotinamide adenine dinucleotide with hydride processes) [38]. Together, these omics data suggest that metabolic disturbances are a feature of geographic atrophy in age-related macular degeneration. This highlights the effectiveness of multiomics when determining disease etiology, an important stepping stone to disease treatment. Uveitis has also been subject to multiomics analysis. Cai et al [39] examined publicly available Gene Expression Omnibus datasets of patients with ankylosing spondylitis and uveitis. A total of 481 shared upregulated genes and 216 shared downregulated genes were found between the 2 cohorts. Upregulated genes were mainly involved in guanosine triphosphatase regulation and protein polyubiquitination. Downregulated genes took part in ribosome biogenesis and oxidative phosphorylation [39].

The available literature on biomarkers in uveitis is limited. Most studies focus on select groups of uveitis subtypes, for example, Behçet disease. This scoping review aims to systematically map the current knowledge on biomarkers in uveitis, without limiting to specific uveitis entities. Biomarkers to be studied include cytokines, chemokines, multiomic data, and microRNAs.

Levac et al [40] established a 6-step framework for conducting scoping reviews. We will use this, alongside the Joanna Briggs Institute (JBI) guidelines, to carry out this research [41]. The aim of this review is to identify what biomarkers have been studied in uveitis, with particular focus on microRNAs, and to guide the selection of biomarkers that may be of interest in future studies.

### Study Objectives

To our knowledge, the most recent systematic review on microRNAs as biomarkers of uveitis was carried out in 2019 by Pockar et al [42]. Of the 20 studies, 8 that were included in the final review were conducted on animals. In addition, this review did not include other molecular biomarkers. While understanding the broader biomarker landscape could provide essential context for identifying specific microRNA signatures, the extent and nature of existing evidence on these other biomarkers remain unclear. No scoping reviews on the topic of microRNAs in uveitis have been conducted to date. Therefore, there is a need to re-examine the literature in a systematic manner to identify established microRNA and other molecular biomarker signatures in uveitis and inform future work, thus driving this scoping review. The aims of the review are as follows: (1) to identify molecular biomarkers in various uveitis subtypes, with particular focus on microRNAs, using the uveitis subtypes defined by the SUN working group to guide the process; (2) to synthesize the available literature in the form of a scoping review; and (3) to identify gaps in knowledge to guide future clinical research projects.

### Research Questions

The JBI Manual details a structured framework for building effective research questions [41]. Their population, concept, and context (PCC) mnemonic can be broken down as follows: population—group of interest to the scoping review; concept—the core concept examined by the scoping review; and context—setting, culture, location, and specific racial or gender-based interests.

For this scoping review, the research questions can be formulated as follows:

- Population—individuals diagnosed with any of the subtypes of uveitis, as per the SUN working group criteria
- Concept—differential expression of molecular biomarkers, such as cytokines, chemokines, microRNAs, and multiomics in biological samples obtained from the population group, compared to healthy controls, with a focus on microRNAs
- Context—this review will not be restricted by geography, gender, race, or location setting

The FINER (feasible, interesting, novel, ethical, and relevant) criteria provide a framework for appraising the value, usefulness, and achievability of research questions [43]. How our research question fits this framework is highlighted in Multimedia Appendix 1.

This leads to the following research question: “What molecular biomarkers have been identified in patients diagnosed with any uveitis entity?”

Arksey and O’Malley [44] provided a 5-stage framework for conducting scoping reviews in 2005. This was later updated to a 6-stage process by Levac et al in 2010 [40]. This subsequent framework will be used alongside JBI guidelines and the PRISMA-ScR (Preferred Reporting Items for Systematic Reviews and Meta-Analyses Extension for Scoping Reviews) checklist to support this scoping review process [41,45]. These stages include (1) identifying the research question; (2) identifying relevant studies; (3) study selection; (4) charting the data; (5) collating, summarizing, and reporting the results; and (6) consultation with stakeholders (optional stage) [40]. A completed PRISMA-ScR checklist for this review is provided in Multimedia Appendix 2.

## Methods

### Identifying Relevant Studies

Scoping reviews can be used to address broader topics, as compared to systematic reviews, which typically focus on a select area with well-defined criteria. In addition, systematic reviews assess the quality of selected research studies, a component that is not strictly necessary for conducting a scoping review [44]. Given the broad nature of scoping reviews, Levac et al [40] highlight the importance of balancing feasibility with breadth and comprehensiveness. They recommend that the review be guided by a team with expertise in the field, to enable a practicable approach that does not compromise the ability of the research question to be answered [40]. This review is being led by the Ocular Immunology Research Group (OIRG) at the Royal College of Surgeons in Ireland (RCSI), which has significant expertise in inflammatory disorders of the eye.

The protocol for this scoping review was registered with the Open Science Framework (OSF) Registries [46].

Studies to be included in this scoping review will be selected from a variety of databases, including MEDLINE, Embase, and Scopus. Databases will be searched using Boolean operators, such as “OR” and “AND.” An example search strategy for the MEDLINE database, conducted on the Ovid search platform on October 15, 2025, is presented in Table 1; this search returned 1656 results when limited to the English language. No date filters were set. The completed PRISMA-S (Preferred Reporting Items for Systematic Reviews and Meta-Analyses literature search extension) checklist is provided in Multimedia Appendix 3 [47]. Expertise from KRW, an information specialist at the RCSI Library, will be sought to guide the search strategy for the formal review process, including confirmation of appropriate databases for inclusion and optimization of the search strategy.

**Table 1:** Example search strategy for the MEDLINE database conducted on October 15, 2025.

	Terms	Papers, n
1	(((“MicroRNAs”[tw] OR “Circulating MicroRNA”[tw] OR “RNA, Small Interfering”[tw]) OR (“Biomarkers”[tw] OR “Endophenotypes”[tw]))] OR [“Cytokines”[tw] OR “Receptors, Cytokine”[tw] OR “Chemokines”[tw]))	1,410,422
2	(“Uveitis”[tw] OR “Uveitis, Intermediate”[tw] OR “Uveitis, Posterior”[tw] OR “Uveitis, Suppurative”[tw] OR “Uveitis, Anterior”[tw] OR “Blau syndrome” [tw] OR “Tubulointerstitial nephritis and uveitis” [tw] OR “Ophthalmia, Sympathetic”[tw] OR “White Dot Syndromes”[tw])	30,847
	1 AND 2	1656

### Study Selection

The results of the search from the included databases will be exported to Covidence (Veritas Health Innovation Ltd), a web-based software platform allowing collaborative selection of papers for reviews of a systematic nature. This software automatically removes duplicates upon importing results. Levac et al [40] identified several challenges associated with conducting a scoping review as a solo endeavor. These include uncertainty about which articles to include, what data to extract from studies, and what to represent in tables during the data charting process. As a result, 2 reviewers will independently screen paper titles and associated abstracts and assess suitability against predetermined inclusion and exclusion criteria. The second reviewer will screen 20% of the papers, with a third reviewer available to resolve discrepancies. Detailed information regarding the inclusion and exclusion criteria is presented in Textbox 1 [48]. These criteria will be revised according to an iterative approach throughout the screening process. Remaining articles will undergo full-text screening and be assessed against the inclusion and exclusion criteria by 2 reviewers, with the second reviewer screening 20% of the papers. This will determine the final articles for inclusion in the scoping review. The reference lists of these papers will be searched for additional relevant studies.

**Inclusion and exclusion criteria.**

**Inclusion criteria**

- Human participants with a diagnosis of uveitis
- Any age
- Any gender
- Any ethnicity
- Any country
- Any health care setting
- Assessment of biomarkers in patients with uveitis, including but not limited to the following: microRNAs, cytokines, chemokines, human leucocyte antigen types, gene variants, and multiomics
- Any sample type, including but not limited to the following: blood, peripheral blood mononuclear cells, aqueous humor, vitreous humor, cerebrospinal fluid, and urine
- Any study type, including but not limited to the following: primary research studies, literature reviews, and systematic reviews
- Articles in English or with an English translation
- Published during any year

**Exclusion criteria**

- Animal participants
- Studies in which the primary focus is on extraocular manifestations of diseases that may cause uveitis
- Non-English papers

### Charting the Data

The results of the included articles will be extracted, and a narrative summary will explain how these results compare to the objective of the scoping review [49]. Research gaps will also be presented.

Microsoft Word and Microsoft Excel will be used to create a template for charting extracted data from the final studies. Peters et al [48] highlight how the information provided in the charting process should take into account the aims of the scoping review. With this in mind, the following data will be extracted and charted, with iterative refinement of these parameters to be undertaken by reviewers during the determination of the final articles for inclusion: title, author, year, location or setting, type of study, sample size, control group size, mean age of participants, uveitis subtypes included and sample sizes, sample type (eg, peripheral blood and aqueous humor), molecular biomarkers studied, microRNA quantification, statistically significant differentially expressed biomarkers compared to control, and additional interesting observations. Comparisons will be drawn between molecular biomarkers expressed in the different uveitis subtypes. Once final studies for inclusion have been identified, 2 reviewers will independently chart data from the first 5 to 10 studies and then meet to collaboratively refine the data extraction and charting process, as recommended by Levac et al [40].

### Summarizing and Reporting the Results

Descriptive analysis best serves the objectives of a scoping review [45]. A narrative assessment of the final studies for inclusion will be provided. Levac et al [40] divide this section into 3 parts: analysis, reporting of results, and applying meaning to the results. A numerical summary will be provided to describe the final included articles. This will detail the total number of included studies, number of study types, years of publication, population demographics, and context [40,44]. Biomarkers differentiating uveitis subtypes will be highlighted. Practical implications of the findings will be discussed, guiding future systematic reviews and primary research studies. Strengths and limitations or gaps of the literature will be identified through this process.

### Pilot Search

A pilot search was carried out to determine the approximate number of studies that will be included in the data extraction process. The MEDLINE database was searched according to the search strategy detailed above. This returned 1656 results when limited to the English language. These papers were ordered in terms of date published, and the top 50 most recent articles were selected for screening. These papers were uploaded to the Covidence platform, and a single reviewer screened the title and abstracts against the inclusion and exclusion criteria. In total, 18 papers were selected for full-text review. Of these, 8 met the inclusion and exclusion criteria for data extraction. This was an iterative process. The inclusion and exclusion criteria were refined during this stage.

The following changes were made to the inclusion and exclusion criteria. We removed the exclusion criterion “studies in which the primary focus is on extraocular manifestations of diseases that may cause uveitis.” This allowed articles that may detail biomarkers of uveitis as secondary objectives to be included. We added “molecular biomarkers” to the inclusion criteria rather than “biomarkers.” The focus of this review will be on molecular biomarkers to guide future primary research in this area. Imaging techniques will be excluded as a result. We excluded studies focusing on therapeutics in the management of uveitis. The focus should be on biomarker discovery. Studies that highlight molecular biomarker changes in response to therapeutics will be included. We added a requirement that studies must have a defined uveitis subtype. The aim of this review is to distinguish uveitis subtypes based on molecular biomarkers, thus making it important for included articles to detail the uveitis subtypes included in their study. We added review articles and letters to the editor to the exclusion criteria. These will be excluded to focus on primary research and for feasibility concerns. We excluded research in progress because such studies do not provide results on molecular biomarkers in uveitis.

## Results

Funding for this study was awarded by the Royal Victoria Eye and Ear Hospital Research Foundation on April 9, 2026. The project start date was January 6, 2026. The 8 studies that met the inclusion and exclusion criteria were then subjected to the data extraction process. A Microsoft Excel worksheet was prepared for this purpose (Multimedia Appendix 4). Data extracted included paper title, author, year of publication, location, study type, sample size (n), sample mean age, control size (n), uveitis subtypes (n), sampling type, biomarkers, microRNAs, biomarker differential expression, and other interesting observations.

Of the 50 articles initially selected, 8 (16%) proceeded to the data extraction phase. If this proportion is extrapolated to all studies identified through the MEDLINE database search, 264 papers will be expected to be included in the scoping review from the MEDLINE database. It is predicted that most articles will come from the MEDLINE database for this review question. A timeline for study completion is presented in Figure 1. As of June 12, 2026, the final search strategy across the MEDLINE, Embase, and Scopus databases has been finalized, and screening of the 946 identified papers is underway using Covidence software.

**Figure 1:**
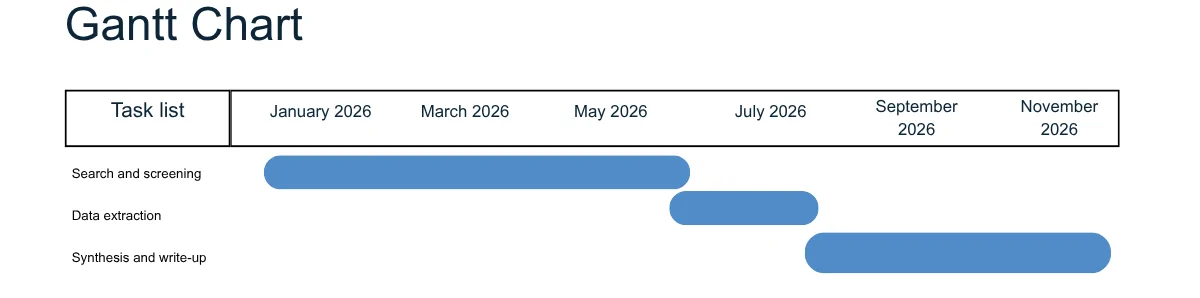
Timeline for scoping review completion.

## Discussion

The aim of this scoping review is to identify current knowledge on molecular biomarkers, particularly microRNAs, in various uveitis subtypes and to guide future primary research studies in this area. We aim to highlight gaps in the literature within this topic, as well as use information gleaned from this review to guide biomarker selection for primary research within the OIRG at the RCSI and The Royal Victoria Eye and Ear Hospital, Ireland. Comparisons can then be drawn to prior work, for example, the previously mentioned systematic review on microRNAs in uveitis by Pockar et al [42]. Results of this review may be disseminated in open-access scientific journals and scientific meetings, appropriate to the subject matter of this research.

Strengths of this scoping review include the breadth of molecular biomarkers examined across uveitis subtypes and its potential to contribute to the development of a panel of molecular biomarkers that may have clinical utility in the diagnosis of this complex disease. In addition, all subtypes of uveitis will be included. Study limitations include the focus on molecular biomarkers of uveitis. Other types of biomarkers, such as imaging biomarkers, will be excluded for feasibility.

Uveitis is a potentially sight-threatening condition. The incorporation of new molecular diagnostic techniques, including microRNA analysis, may expedite diagnosis, facilitate early targeted treatment, and ultimately improve outcomes for patients globally.

## Appendix

Multimedia Appendix 1 FINER (feasible, interesting, novel, ethical, and relevant) criteria.

Multimedia Appendix 2 PRISMA-ScR (Preferred Reporting Items for Systematic Reviews and Meta-Analyses Extension for Scoping Reviews) checklist.

Multimedia Appendix 3 PRISMA-S (Preferred Reporting Items for Systematic Reviews and Meta-Analyses literature search extension) checklist.

Multimedia Appendix 4 Data extraction chart.

## Abbreviations

BEST: Biomarkers, Endpoints, and Other Tools
EAU: experimental autoimmune uveoretinitis
EIU: endotoxin-induced uveitis
FINER: feasible, interesting, novel, ethical, and relevant
GRO-alpha: growth-regulated oncogene-alpha protein
JBI: Joanna Briggs Institute
MCP-1: monocyte chemoattractant protein 1
MIG: monokine induced by gamma interferon
OIRG: Ocular Immunology Research Group
PCC: population, concept, and context
PRISMA-S: Preferred Reporting Items for Systematic Reviews and Meta-Analyses literature search extension
PRISMA-ScR: Preferred Reporting Items for Systematic Reviews and Meta-Analyses Extension for Scoping Reviews
RANTES: regulated upon activation, normal T-cell expressed and secreted
RCSI: Royal College of Surgeons in Ireland
SLE: systemic lupus erythematosus
SUN: Standardization of Uveitis Nomenclature
VKH: Vogt-Koyanagi-Harada

## Data Availability

The data generated or analyzed during this study are available from the corresponding author on reasonable request.
