# Supplementary material for: The Role of MicroRNAs and Other Molecular Biomarkers in Uveitis: Protocol for a Scoping Review
Source: JMIR Res Protoc. 2026 Sep 25;15:e89265. doi: 10.2196/89265 (PMC13653206; doi:10.2196/89265)
Supplement: Supplementary file 2 [file resprot_v15i1e89265_app1.docx]

Supplementary Material 1: FINER Criteria

The FINER criteria define a framework for appraising the value, usefulness, and achievability of research questions[1]. This framework fits our research question as follows:

**F**easibility – The scoping review detailed in this protocol is feasible within the timeframe allotted.

**I**nteresting – Examination of the literature for potential biomarkers of uveitis may help amalgamate study results, in order to develop a diagnostic panel of biomarkers, which may aid in the management of uveitis patients in the future.

**N**ovel – The focus of this study is biomarkers in uveitis, particularly microRNAs as diagnostic biomarkers. As already mentioned, no scoping review has been conducted to date on this topic.

**E**thical – Given the nature of the review process, no ethical concerns arise as no new patient data is being generated.

**R**elevant – Uveitis can present a challenge to ophthalmologists given the large number of subtypes. This scoping review may help to guide development of a diagnostic panel of biomarkers, which could be used to differentiate uveitis subtypes, leading to more rapid diagnoses, and thus reducing patient and economic burden.

1.	FINER: a research framework. Elsevier.: Elsevier.; [cited 2025 13th October 2025]; Available from: https://scientific-publishing.webshop.elsevier.com/research-process/finer-research-framework/.
