# Supplementary material for: The Role of MicroRNAs and Other Molecular Biomarkers in Uveitis: Protocol for a Scoping Review
Source: JMIR Res Protoc. 2026 Sep 25;15:e89265. doi: 10.2196/89265 (PMC13653206; doi:10.2196/89265)
Supplement: Supplementary file 4 [file resprot_v15i1e89265_app4.docx]

**Data Extraction and Charting Form**

| Title | Author | Year | Country | Study Type | Sample Size (n) | Sample Mean Age | Control Size (n) | Uveitis Subtype/s (n) | Sample Type | Biomarkers | MicroRNA Expressed | Notes |
| --- | --- | --- | --- | --- | --- | --- | --- | --- | --- | --- | --- | --- |
|  |  |  |  |  |  |  |  |  |  |  |  |  |
|  |  |  |  |  |  |  |  |  |  |  |  |  |
|  |  |  |  |  |  |  |  |  |  |  |  |  |
|  |  |  |  |  |  |  |  |  |  |  |  |  |

The following charting form was developed iteratively in accordance with Joanna Briggs Institute guidance for scoping reviews and was piloted and refined by the review team prior to full data extraction.
